# Salt concentration and charging velocity determine ion charge storage mechanism in nanoporous supercapacitors

**DOI:** 10.1038/s41467-018-06612-4

**Published:** 2018-10-08

**Authors:** C. Prehal, C. Koczwara, H. Amenitsch, V. Presser, O. Paris

**Affiliations:** 1 Institute of Physics, Montanuniversitaet Leoben, Franz-Josef Straße 18, 8700 Leoben, Austria; 2 Institute of Inorganic Chemistry, Graz University of Technology, Stremayrgasse 9/IV, 8010 Graz, Austria; 3 INM-Leibniz Institute for New Materials, Campus D2 2, 66123 Saarbrücken, Germany; 4 Department of Materials Science and Engineering, Saarland University, Campus D2 2, 66123 Saarbrücken, Germany; 5 Present Address: Institute for Chemistry and Technology of Materials, Graz University of Technology, Stremayrgasse 9/V, 8010 Graz, Austria

## Abstract

A fundamental understanding of ion charge storage in nanoporous electrodes is essential to improve the performance of supercapacitors or devices for capacitive desalination. Here, we employ in situ X-ray transmission measurements on activated carbon supercapacitors to study ion concentration changes during electrochemical operation. Whereas counter-ion adsorption was found to dominate at small electrolyte salt concentrations and slow cycling speed, ion replacement prevails for high molar concentrations and/or fast cycling. Chronoamperometry measurements reveal two distinct time regimes of ion concentration changes. In the first regime the supercapacitor is charged, and counter- and co-ion concentration changes align with ion replacement and partially co-ion expulsion. In the second regime, the electrode charge remains constant, but the total ion concentration increases. We conclude that the initial fast charge neutralization in nanoporous supercapacitor electrodes leads to a non-equilibrium ion configuration. The subsequent, charge-neutral equilibration slowly increases the total ion concentration towards counter-ion adsorption.

## Abstract (web summary)

To improve supercapacitor performance, mechanisms of operation should be understood. Here the authors identify parameters controlling ion charge storage mechanisms and show that charging initially implies a non-equilibrium ion configuration followed by charge-neutral equilibration.

## Introduction

The interactions between ions, solvent molecules, and the internal surface of an electrically conductive, nanoporous electrode material determine ion electrosorption mechanisms and their related phenomena^1–4^. The request for further increasing the performance of supercapacitors and devices for capacitive deionization (CDI) demands a fundamental, microscopic understanding of both equilibrium and dynamic behavior of ion charge storage^1,5^.

When carbon-based supercapacitors are charged, the (non-Faradaic) electrode charge is counter-balanced by the ionic charge within the pore space. At the potential of zero charge (PZC), the number of cations and anions within the pores is balanced. Upon charging, there are three modes for charge-balancing: the adsorption of additional counter-ions (counter-ion adsorption), the desorption of co-ions (co-ion expulsion), or the concurrence of counter-ion adsorption and co-ion desorption (ion replacement or ion swapping)^3,5^. The charging mechanism is typically characterized by either identifying the difference between counter-ion and co-ion concentration at a certain electrode charge^3,6^ or the derivative of the latter, that is, the change of counter- and co-ion concentrations with increasing electrode charge^7^.

Cation and anion concentration changes during charging can be measured by different experimental methods like in situ nuclear magnetic resonance (NMR)^6^, electrochemical quartz crystal microbalance (eQCM)^8^, or in situ X-ray transmission (XRT) measurements^9^. In situ small-angle X-ray scattering (SAXS) and atomistic modeling^10,11^ have shown that in addition to concentration changes, there is local ion rearrangement across the nanopores combined with partial desolvation. Ions rearrange to optimally screen repulsive interactions between counter-ions by preferentially occupying sites with highest possible degree of confinement^12^. This mechanism naturally explains the often reported increase of surface-normalized capacitance with decreasing micropore size^13,14^. Spectroscopic techniques^6,15^ allow the effective measurement of concentration changes of specific chemical species within the system. By use of XRT, both cation and anion concentration changes can be quantified at the same time and correlated to the electrode charge^16^. Key advantages of in situ XRT are the simple experimental setup, the high time resolutions, and the flexibility of cell designs. So far, ion replacement^6,9^, counter-ion adsorption^7,17,18^, and to some extend co-ion expulsion^6^ have been observed during ion electrosorption in organic and aqueous electrolytes. While eQCM experiments^7,8,18,19^ preferentially obtained counter-ion adsorption for a number of different systems, in situ NMR^6,20,21^ and in situ XRT^9,10^ studies typically indicate the dominance of ion replacement. However, experimental conditions and key-parameters determining the dominating ion charge storage mechanism still remain to be identified. Both atomistic/molecular parameters, such as carbon/ion interactions, ion mobilities or hydration enthalpies, and macroscopic properties of the entire system, like cell design or cycling rates, might ultimately influence the charge storage mechanism in a yet unknown way.

Here we present a systematic investigation of ion electrosorption mechanisms in a microporous activated carbon-based electrical double-layer capacitor (EDLC) using aqueous electrolytes with different salt concentrations (details of all materials used, see Methods section). In situ XRT and small-angle X-ray scattering experiments during charging and discharging in a custom-built supercapacitor cell^16^ reveal distinct dependencies of the ion charge storage mechanism on the electrolyte salt concentration, the charging and discharging rates, the specific cell design and partially the nature of the used ions. Cation and anion concentration changes are discussed based on cyclic voltammetry (CV) data at four different scan rates. Varying the type of ions, and thus the sensitivity of the X-ray transmission of cations and anions, provides compelling evidence for the strong dependence of the storage mechanism on ion concentration, cycling speed, and cell design. Moreover, changes of cation and anion concentrations on time scales much larger than the time of the actual charging were detected during chronoamperometry (CA) measurements, suggesting that the first fast time regime does not lead to the final equilibrium configuration of the system.

## Results

### Electrochemical characteristics

**Fig. 1:**
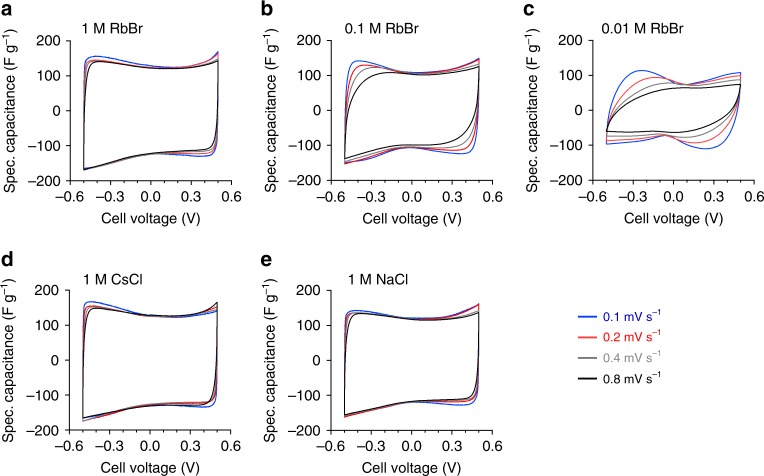
Cyclic voltammograms of all investigated in situ cells. Specific capacitance versus cell voltage for the in situ cells using the same activated carbon as working electrode (WE) material and different aqueous electrolytes. 1 M RbBr (**a**), 0.1 M RbBr (**b**), 0.01 M RbBr (**c**), 1 M CsCl (**d**), and 1 M NaCl (**e**) tested at four different scan rates (0.1–0.8 mV s^−1^) with ± 0.5 V applied cell voltage

Cyclic voltammograms (corrected for leakage currents, see Supplementary Fig. 1, Supplementary Note 1) of in situ cells using aqueous 1, 0.1, and 0.01 M RbBr electrolyte (Fig. 1a–c) reveal differences in the capacitance and its voltage dependence. CV curves of cells with the lowest salt concentration tend to show a distinct minimum around the potential of zero charge (PZC) at low scan rates. For high molar electrolytes, such butterfly-shape is often referred to the capacitance contribution of the carbon electrode, which depends on the voltage-dependent electronic charge carrier density at the Fermi level^22–24^. In most nanoporous carbon materials, this “space charge” contribution *C*_sc_ from the carbon material is not negligible compared to the contribution from the Helmholtz layer *C*_H_ and the diffuse layer $C_{\mathrm {diff}}\left( {\frac{1}{{C_{\mathrm {total}}}} = \frac{1}{{C_{\mathrm {SC}}}} + \frac{1}{{C_{\mathrm {H}}}} + \frac{1}{{C_{\mathrm {diff}}}}} \right)$. Within those models, the voltage dependence reflects the electronic density of states of the carbon material and is referred to as quantum capacitance. However, in the present work a distinct minimum around the PZC was not only visible at high concentrations but was even more pronounced for low molar electrolytes (Fig. 1c). It is therefore most probably caused by the capacitance contribution of the diffuse layer *C*_diff_, as predicted by the Gouy–Chapman–Stern (GCS) theory in low-concentration electrolytes^25,26^. The kinetic offset between the capacitance minimum in Fig. 1c during charge and discharge is largely induced by the limited ionic conductivity in low molar electrolytes. Moreover, the particular design of the in situ cell^16^ enlarges the diffusion pathways for ions diffusing from one electrode to the other (compared to, for example, a standard cell assembly in a Swagelok cell), also being detrimental for a good rate performance.

Apart from the minimum around the PZC, the decrease in capacitance with lower molarities is well seen at higher scan rates. This effect is caused by the lowered ionic conductivity, and reflects the limited power handling of the system under these conditions. 1 M CsCl and 1 M NaCl cells show, compared to 1 M RbBr, neither a clear difference in capacitance nor in the shape of the CV curves (Fig. 1d, e). In addition, gravimetric capacitance, cyclic stability, and rate handling of 1 M RbBr, 1 M CsCl, and 1 M NaCl electrolytes were determined in a symmetric custom-built cell, optimized for supercapacitor performance testing^27^ (Supplementary Fig. 2, Supplementary Note 2). The specific capacitance at lower charging/discharging currents is practically equal for all three electrolytes and remains stable for at least 1000 cycles. A detailed discussion regarding the specific selection of electrolytes is given in Supplementary Note 3.

### In situ X-ray transmission

In situ XRT allows quantifying ion concentration and corresponding changes during charging and discharging of nanoporous carbon supercapacitor electrodes^9,16^. The X-ray transmission τ is defined as the ratio between transmitted intensity of the (primary) X-ray beam and the incident beam intensity. According to Lambert–Beers law, the X-ray intensity decays exponentially with sample thickness *d* when penetrating a material (*τ=*exp(*−μd*)). The linear attenuation coefficient *μ* is a material-specific parameter and depends also on the energy of the incident X-ray photons. The negative logarithm of the transmission *τ* for the in situ supercapacitor cell corresponds to the sum of cation and anion concentrations (*c*_cat_, *c*_an_) weighted by their respective mass attenuation coefficient $\left( {\frac{\mu }{\rho }} \right)$, their molar mass *M* and the electrolyte “thickness” *d*_el_ in beam direction. Additionally, it includes a term accounting for the solvent (water) absorption (with *ρ*_H2O_ being the water mass density) and a constant term considering the solid phases in the beam (carbon, current collector, separator, and tape windows)^16^.

1 $$- \ln \left( \tau \right) = \left[ {c_{\mathrm{cat}}{M}_{\mathrm {cat}}\left( {\frac{\mu }{\rho }} \right)_{\mathrm{cat}} + {c}_{\mathrm{an}}{M}_{\mathrm{an}}\left( {\frac{\mu }{\rho }} \right)_{\mathrm{an}} + \rho _{\mathrm {H}_2{\mathrm{O}}}\left( {\frac{\mu }{\rho }} \right)_{\mathrm {H}_2{\mathrm{O}}}} \right]d_{\mathrm {el}} + \rho _C\left( {\frac{\mu }{\rho }} \right)_Cd_C.$$

Raw transmission data for scan rates of 0.1 mV s^−1^ using cells with 1 M CsCl, 1 M RbBr, 1 M NaCl, 0.1 M RbBr and 0.01 M RbBr are shown in Supplementary Fig. 3. To obtain cation and anion concentration changes independently from each other, the electrode charge needs to be calculated by integrating the measured current over time^9,16^. In addition, the initial cation and anion concentration within the working electrode (WE) at the PZC must be estimated^16^. In electrolytes with a 1 M salt concentration, the initial concentration within the WE pores should correspond in a good approximation to the bulk concentration of 1 M. For smaller salt concentrations, however, image forces of the conducting electrode attract both sorts of ions, resulting in an increased concentration within the nanopores, even at zero applied voltage^28^. Since the absolute value of the transmission signal (Eq. 1) contains contributions from all species in the irradiated volume, like carbons atoms, water molecules as well as cations and anions both in micropores and the bulk electrolyte in the macropores, the initial micropore ion concentration at the PZC is experimentally difficult to access.

Therefore, the ion concentration change is visualized here by plotting the negative logarithm of the transmission signal subtracted by its value at zero electrode charge In (*τ*_0_) as a function of the electrode charge (Eq. 2).

2 $$A = - \ln \left( \tau \right) + \ln \left( {\tau _0} \right) = \left[ {\Delta c_{\mathrm {cat}}{M}_{\mathrm {cat}}\left( {\frac{\mu }{\rho }} \right)_{\mathrm {cat}} + \Delta c_{\mathrm {an}}{M}_{\mathrm{an}}\left( {\frac{\mu }{\rho }} \right)_{\mathrm {an}} + \Delta \rho _{\mathrm {H}_2{\mathrm{O}}}\left( {\frac{\mu }{\rho }} \right)_{\mathrm {H}_2{\mathrm{O}}}} \right]d_{\mathrm {el}}.$$

**Fig. 2:**
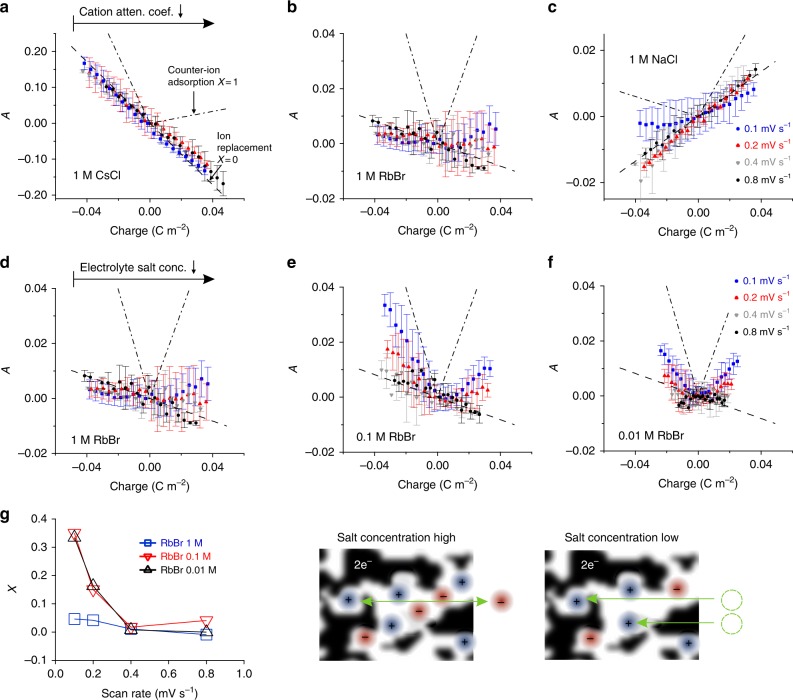
Quantification of parameters controlling ion charge storage mechanisms. **a**–**f** Relative attenuation *A* (Eq. 2) vs. electrode charge for in situ cells using different aqueous electrolytes at four different scan rates with ±0.5 V maximum applied cell voltage. In **a**–**c**, the attenuation coefficient of cations (Table 1) becomes smaller from left to right, whereas in **d**–**f**, the salt concentration was decreased from left to right. The black lines indicate the theoretical attenuation curves for pure ion swapping (dashed) and counter-ion adsorption (dashed dotted) calculated from Eq. 2. Note the different scales in **a**–**c**. The charge storage mechanism is quantified in **g** plotting the charge storage parameter *X* (see Eq. 3) vs. the scan rate for RbBr. With decreasing salt concentration counter-ion adsorption becomes the dominant charge storage mechanism, as visualized in **g** on the right

Using this approach, the curves in Fig. 2 can be understood as the sum of cation and anion concentration change weighted by their corresponding effective mass attenuation coefficients (and *d*_el_). We refer to the parameter *A*=−ln(*τ*)+ln(*τ*_0_) as (relative) X-ray attenuation. The representation of the parameter *A* in Fig. 2 is therefore in full analogy with the usual visualization of cation and anion fluxes from eQCM experiments^7^, and can be interpreted in the same manner.

The average density of water within the pores (i.e., the third term in Eq. 2) might also change during charging and discharging. The ions are covered by hydration shells with different water density. If the concentration of the specific ions is changing during charging, the average water density is also changing. Another reason for water densification might be an increased osmotic pressure if ion concentrations increase during charging. However, ion concentrations do not exceed 1 M and the ions are of similar size. Consequently, the influence of the water term in Eq. 2 is expected to be small and can be neglected on first approximation in this work.

To experimentally observe possible counter-ion adsorption for electrolytes with lower salt concentrations, the cell assembly had to be re-designed as compared to previous experiments^9,16^. The electrolyte reservoir was strongly enlarged (partially to avoid ion starvation^29^ in low molar electrolytes), and the WE mass was kept as small as possible. Only such large bulk electrolyte volume (about a factor of 100 larger than the WE micropore volume) ensures that the mean bulk electrolyte concentration change will be negligible during possible counter-ion adsorption when the total ion concentration increases in both electrodes.

### In situ X-ray transmission during cyclic voltammetry

Cells with 1 M CsCl, 1 M RbBr, and 1 M NaCl show very similar electrochemical response (Fig. 1a, d, e), but a notably different X-ray attenuation behavior (Fig. 2a–c). These salts with their strongly different ratios of cation and anion attenuation coefficients (Table 1) enable a consistent interpretation of the experimental data using Eq. 2 (detailed discussion see Supplementary Note 3). Thus differences in the experimental data of the different salts are mainly induced by the different X-ray attenuation coefficients and not by differences regarding the ion charge storage mechanism or the electrochemical performance^9^. This “contrast variation” is an additional source of information and proves the general validity of the experimental approach and the corresponding model in Eq. 2. The predictions for pure counter-ion adsorption (black, dashed dotted line in Fig. 2a–f) and pure ion swapping (black dashed line) are based on Eq. 2 using the charge as input. Pure ion swapping corresponds to a linear behavior for all (both positive and negative) values of electrode charge, while counter-ion adsorption is related to a deviation from this level towards more positive values. According to Fig. 2a–c, large salt concentrations of 1 M show almost pure ion swapping as the main charging mechanism in nanoporous carbon electrodes.

**Table 1:** Effective (molar) attenuation coefficients, bare (crystal) ion radii, hydration enthalpies and ion mobilities for Cs^+^, Cl^−^ Rb^+^, Br^−^, and Na^+^ ions in aqueous solution

	Molar attenuation coefficient^35^ ${\mu}_{{\bf eff,ion}} = {{\bf M}}_{{{\bf ion}}}\left( {\frac{{{\mu }}}{{{\rho }}}} \right)_{{\bf{ion}}}$ (10^-3^ mol^−1^ cm^2^)	Bare ion radius (nm) after ref. ^36^	Hydration enthalpy (kJ mol^−1^) after ref. ^25^	Ion mobility (10^−8^ m^2^ V^−1^ s^−1^) after ref. ^37^
Cs^+^	43.7	0.169	−17.1	7.3
Cl^-^	3.85	0.181	−19.5	6.9
Rb^+^	9.2	0.148	−18.5	7.7
Br^-^	7.3	0.195	−17.9	7.2
Na^+^	0.7	0.095	−24.5	5.0

At smaller salt concentrations and low scan rates, the counter-ion adsorption becomes more dominant (Fig. 2d–f). Notably, at larger scan rates ion swapping was observed for 0.1 M and even for 0.01 M concentrations.

It has to be noted that the theoretical prediction for pure counter-ion adsorption may not be perfectly reached by any system, since an increase of the total ion concentration within the pores should always be accompanied by a release of some water molecules and thus by a decrease of the relative attenuation *A*. In addition, to some degree ion swapping (or permselectivity failure), is always present close to the PZC^7^, making the theoretical curves for counter-ion adsorption hard to reach.

Equivalently to the point of zero mass change in eQCM data^7^, the minima in Fig. 2e, f might be attributed to the point of zero attenuation change (PZAC). In the case that cation and anion attenuation coefficients are similar, the PZAC might be close to the PZC. However, considering the relatively large errorbars in Fig. 2 and the influence of the actual, prevalent ion charge storage mechanism the PZAC can be only seen as a rough estimate for the position of the PZC.

In order to parametrize the charging mechanism with a single number, Forse et al^3^. have introduced the charging mechanism parameter *X*, which is +1 for pure counter-ion adsorption, 0 for pure ion swapping and −1 for pure co-ion expulsion. We calculate *X* for an electrode charge *Q* of 0.02 C m^−2^ via Eq. 3:

3 $${X}\left( {Q} \right) = \frac{{A_{\mathrm {meas}}\left( Q \right) - A_{\mathrm {theo}}^{\mathrm {swapp}}(Q)}}{{A_{\mathrm {theo}}^{\mathrm {counter}}(Q) - A_{\mathrm {theo}}^{\mathrm {swapp}}(Q)}}.$$

As an alternative definition of *X(Q)* the derivative d*A(Q)/*d*Q* could be used instead of *A(Q)* in Eq. 3. This would reflect the mass transport of counter-ions and co-ions in and out of the electrode at a certain charge, where the definition of the charging mechanism in this work is based on the total counter-ion and co-ion concentration in the pores at a specific charge. The latter is equivalent to the definition in ref. ^3^.

The average of *X(Q)* at negative and positive polarization (at ±0.02 C m^−2^) as a function of the scan rate (Fig. 2g) shows a distinct dependence of the charging mechanism on the scan rate and the salt concentrations. For cell designs used in this work and for large enough scan rates (≥0.4 mV s^−1^), ion swapping dominates for all salt concentrations investigated. However, at very small scan rates and for low salt concentrations, counter-ion adsorption tends to become competitive. This finding strongly suggests that the detailed mechanism (ion swapping or counter-ion adsorption) does not only depend on salt concentration, but also on the scan rate. A transition from ion swapping towards counter-ion adsorption is clearly observed if the scan is performed slow enough. Thus, we conclude that a simple exchange of counter- and co-ions observed for large scan rates even for very low salt concentrations must correspond to a non-equilibrium or transient state of cation and anion concentrations within the pores.

Besides the scan rate dependence (i.e., the kinetic behavior of the system), the equilibrium ion concentration is influenced by the salt concentration of the bulk electrolyte. Consequently, we expect also a dependence of the ion charge storage mechanism on the specific assembly of the EDLC cell. Cell designs with relatively small bulk electrolyte volume should suppress counter-ion adsorption, as supported by XRT data using an alternative cell assembly (Supplementary Fig. 4 and related discussion^28^ in Supplementary Note 4). This finding is of high relevance for the experimental characterization of supercapacitors or related technologies like CDI. Our data indicate that not only the comparability between different in situ techniques (all using very specific cell designs), but also between sophisticated experiments and commercial devices is limited. While in many eQCM studies^7,17,18^, a tendency toward counter-ion adsorption can be observed even for high molar electrolytes, the finite electrolyte reservoir in in situ XRT/SAXS^9^ or NMR experiments^6^ seems to support ion swapping. Consequently, specific charge storage mechanisms deduced from experimental data on the smallest accessible length scale should be generalized only if cell design, scan rate, and salt concentration are properly considered.

### In situ X-ray transmission during chronoamperometry

**Fig. 3:**
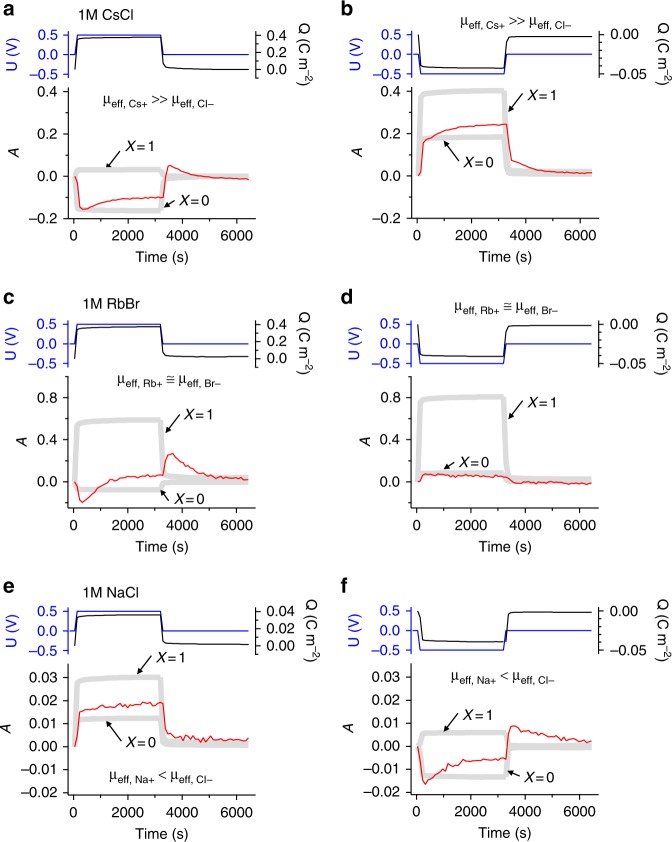
Ion concentration change during potentiostatic charge/discharge. Cell voltage *U* (blue), charge signal *Q* (black), and relative attenuation *A* (red) versus time for 1 M CsCl, 1 M RbBr, and 1 M NaCl for a single chronoamperometry step at + 0.5 V (**a**, **c**, **e**) and -0.5 V (**b**, **d**, **f**). The thick gray lines indicate the theoretical attenuation curves calculated from Eq. 2 for pure ion swapping (*X* = 0) and counter-ion adsorption (*X* = 1). Note the different ratios between cation and anion attenuation coefficients *μ*_*eff*_ of the different salts (Table 1)

Since the ion charge storage mechanism shows a distinct dependence on the CV scan rate even at very low scan rates, it is worthwhile to look more closely at the time dependence of the transmission signal and the relative absorption *A* using chronoamperometry (CA) measurements. At positive polarization, the 1 M CsCl cell (Fig. 3a) shows a fast decline of the relative absorption before it slowly increases again. The gray dashed lines indicate the theoretical curves for pure ion swapping (*X* = 0) and pure counter-ion adsorption (*X* = 1). Similarly, at negative polarization (Fig. 3b) two different regimes of the relative absorption signal can be identified: a fast increase with the same magnitude as the fast decline at positive polarization, and a subsequent slower increase. Notably, global electrode charging had stopped already after very short times (black curve on the top of Fig. 3a, b). This implies that for both polarizations the electrode charge is first counter-balanced via ion swapping by a first, fast process, while on the longer term the ion concentrations change towards counter-ion adsorption.

The 1 M RbBr cell shows for positive polarization again two processes within strongly different time regimes (Fig. 3c). For charging, at first the effective absorption decreases, before it increases again. At negative polarization (Fig. 3d) the effective absorption shows only a very slight increase upon charging and remains rather constant after a short period of time.

The 1 M NaCl (Fig. 3e, f) cell behaves essentially like the 1 M CsCl cell, but with reversed polarization dependence due to the reversed order of the cation and anion absorption strength (see Table 1). The transmission raw-data for all CA measurements, including 0.1 M and 0.01 M RbBr, are given in Supplementary Fig. 5.

To verify whether changes of the effective attenuation can be attributed to changes within the carbon micropores only, in situ SAXS measurements were carried out using the AC electrode and aqueous 0.1 M RbBr (Supplementary Fig. 6 and Supplementary Note 5). Equivalent features of a two-step process (like in Fig. 3) were observed in the time-dependent SAXS intensity in a scattering angle regime covering ion concentration changes from micropores only^9,10,16^.

## Discussion

**Fig. 4:**
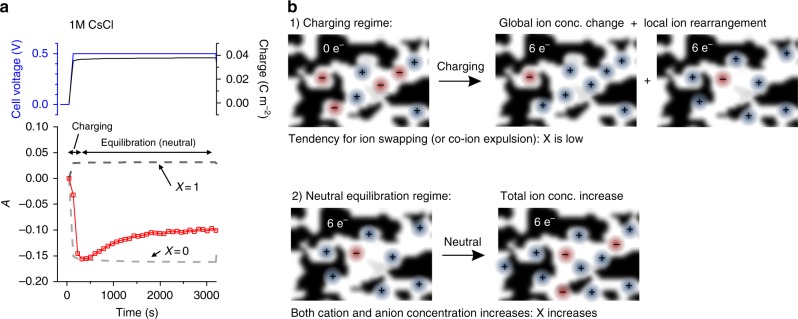
Two distinct time regimes of ion concentration changes. **a** Relative attenuation *A* (red data points), applied cell voltage *U* (blue) and charge *Q* (black) vs. time are shown exemplarily for a single chronoamperometry charging step at +0.5 V (left) for 1 M CsCl. The gray and the black dashed line indicate the theoretical attenuation curves for pure ion swapping (*X*=0) and pure counter-ion adsorption (*X*=1), respectively. As visualized on the right (**b**), ion concentration changes can be separated in two processes occurring at significantly different time scales: a fast charging regime with ion swapping as the main charging mechanism (recorded by in situ X-ray transmission, XRT) in combination with local ion rearrangement (recorded by in situ small angle X-ray scattering, SAXS) and a subsequent neutral increase of the total ion concentration (recorded by in situ XRT) causing a slow transition towards counter-ion adsorption

The observed two-step process shall now be discussed in more detail for the case of the 1 M CsCl cell (Fig. 4a). On a timescale that corresponds to conventional supercapacitor charging times (i.e., seconds to several hundred seconds) the charging mechanism corresponds to pure ion swapping. According to the electrode charge signal the actual charging process has finished after a very short period. On larger time scales, the relative absorption *A* in Fig. 4 increases again, while the electrode charge remains perfectly constant on these time scales. Thus, the increase can only be interpreted as a concentration increase of both cations and anions at exactly the same amount. This means that the net current is zero, but the ratio between counter-ion and co-ion concentration has changed. Effectively, this leads to a transition from ion swapping to counter-ion adsorption. In equilibrium, aqueous 1 M CsCl tends toward counter-ion adsorption for both positive and negative polarization. This implies that the number of co-ions is initially decreasing before it slightly increases again.

At time scales typically used in supercapacitor research, the dominant charge storage mechanism is ion replacement, but ion concentrations in the pores represents only a transient state. We have shown previously^10^, that besides global ion concentration changes, counter-ions preferably move into pore-sites with highest possible geometrical confinement (local rearrangement) and, if necessary, do so by partially striping off their solvation shell (charging regime, Fig. 4b). Features in the in situ SAXS data (Supplementary Fig. 6d) suggest that the local rearrangement mainly takes place in the charging time regime. The slow equilibration process implies only a small gain in the free energy of the system when increasing the total ion concentration (i.e., also changing the ratio of cations and anions), and the net ionic charge remains constant (neutral equilibration regime, Fig. 4b).

While the CsCl and NaCl systems show a very similar behavior (Fig. 3a, b, e, f), RbBr behaves differently. At positive polarization, co-ion expulsion occurs during charging. In the neutral equilibration regime, the total ion concentration increases, causing a transition towards ion swapping and subsequently (partial) counter-ion adsorption (Fig. 3c). At negative polarization, we see ion swapping in the charging regime (Fig. 3d). Here, cation and anion concentrations remain constant after electrode charging has stopped. Since the in-pore ion concentration in the charging regime represents a transient state, diffusive properties of the different ion species might play a significant role. Co-ion expulsion of Br^−^ ions at positive polarization might be attributed to their high ion mobilities and consequently their smaller hydration enthalpies compared to Rb^+^ ions (Table 1). A high Br^−^ mobility would imply a fast expulsion of Br^−^ ions and a slower adsorption of Rb^+^ ions at positive polarization, effectively leading to co-ion expulsion at small timescales.

In summary, the present study demonstrates a clear non-equilibrium behavior of ionic charging in nanoporous supercapacitors even at slow scan rates and for highly conductive aqueous electrolytes. This emphasizes an important issue in supercapacitor research. It is extremely difficult (and requires extremely long charging times) to obtain actual stable equilibrium ion configurations in the nanoporous electrodes of a supercapacitor^30^. In an experimental situation, the behavior of ions on the atomic scale usually must be drawn from non-equilibrium ion configurations that can change significantly by slightly changing, e.g., temperature^30^, the size of the electrolyte reservoir or the salt concentration^5,28^.

Ion charging mechanisms on short time scales, typical for charging carbon-based supercapacitor electrodes, were shown to depend on the ionic strength of the electrolyte, the charging/discharging rate and the specific design of the supercapacitor cell. These dependencies should always be considered when comparing different in situ methods with each other or with simulation studies. So far however, the measured ion charge storage mechanisms have in most studies only been discussed regarding local ion-carbon, ion–solvent and ion–ion interactions. In situ XRT during CA measurements clearly demonstrate that the total ion concentration further increases after the actual charging/discharging of the supercapacitor has stopped. This implies a transition towards higher charging parameter *X* (i.e., counter-ion adsorption) although the difference between adsorbed counter- and co-ions remains equal on these timescales.

Since equilibrium ion concentration within the micropores have typically not been reached after fully charging of the supercapacitor, the actual charge storage mechanism in this time regime may be strongly influenced by kinetic properties of the different ion species. This may imply preferable co-ion desorption, if the co-ion has a high mobility or preferable counter-ion adsorption if the mobility of counter-ions is high. Moreover ion-concentration dependent changes of the ion diffusion coefficient due to mutual ion blocking might play a role^31^.

Further systematic investigations of non-equilibrium properties of the supercapacitors are essential to improve the comparability between fundamental studies using atomistic simulations and the various in situ experiments, enabling further progress in optimizing the performance of commercial devices in the future.

Notably most important parameters influencing ion charge storage mechanisms are properties of the entire system, such as salt concentration, charging velocity or cell design, rather than properties on the molecular scale. Therefore, most relations found in this work should be generally valid and applicable to a wide range of electrode–electrolyte combinations (including organic solvents). Subtle differences regarding the time-dependent charge storage mechanism were induced by the nature of the used ions and should depend on molecular phenomena, such as partial dehydration and the enhancement of the surface normalized capacitance in strong confinement.

## Methods

### The in situ experiment

In situ X-ray transmission (XRT) measurements were carried out on a laboratory SAXS instrument (NanoStar, Bruker AXS) using Cu Kα radiation and a Vantec 2000 area detector^32^. The transmission signal was measured using glassy carbon (GC) as a quantitative standard^33^, where a GC sample is placed in the beam right behind the measurement cell. In a good approximation, the transmission signal corresponds to the total intensity of the in situ cell plus GC measured by the area detector, divided by the integrated intensity of GC alone. While recording such 2D patterns from the working electrode every 90–180 s, CV or CA was applied to the in situ supercapacitor cell via a Gamry Ref600 potentiostat.

Since the photon flux of the laboratory X-ray source was too low to perform in situ SAXS experiments at higher charging rates, we used the Austrian SAXS beamline at the synchrotron radiation source ELETTRA (Trieste, Italy) to collect the SAXS data shown here. Measurements and data analysis was performed following the experimental setup and protocols described previously^9,10,16^.

All in situ XRT and SAXS measurements were performed with a custom-built in situ supercapacitor cell^16^. Holes of 6 mm diameter in the titanium and polyether ether ketone (PEEK) housing are sealed with tape, which ensures the almost undistorted transmission of the X-ray beam. The cell assembly used thin (ca. 200 nm) platinum paper as current collector (CC), an activated carbon (AC) working electrode (WE), an AC counter electrode (CE), and a Whatman GF/A glass fiber separator in-between. To provide a sufficiently large electrolyte volume for cells with low salt concentrations, five separator layers were stacked on top of each other. The asymmetric cell design (CE oversized by a factor of 15 in volume) guaranteed that the current is limited by the WE and almost the entire applied cell voltage drops at the WE. A hole of 3 mm in diameter in the CE (and in 4 out of 5 separators) ensured large enough transmission and warranted changes of the transmission and scattering signal originating from the WE only. The WEs had a diameter of 6 mm, a thickness of 200 ± 15 µm and a mass of 3.2 mg.

### Materials

WEs were prepared by mixing the AC powder (MSP20, Kansai Coke and Chemicals) with ethanol and 10 mass% of dissolved polytetrafluoroethylene (PTFE, 60 mass% solution in water from Sigma Aldrich) in a mortar^22^. The material was rolled with a rolling machine (MSK-HRP-MR100A, MTI) to a 200 ± 15 µm thick free-standing film electrode and dried at 120 °C at 2 kPa for 24 h. Gas sorption analysis of the WE was performed using N_2_ and CO_2_ sorption of the AC electrode. Data analysis by quenched solid density functional theory^34^ revealed a specific surface area of 1707 m^2^ g^−1^ and an average pore size of 0.9 nm, as already reported in previous work^16^.

We used as electrolytes aqueous solutions of RbBr at concentrations of 1, 0.1, and 0.01 M; as well as CsCl and RbBr at concentrations of 1 M.

## Electronic supplementary material

Supplementary Information

## Data Availability

XRT raw data were generated at a laboratory SAXS instrument. SAXS raw data were generated at the large-scale synchrotron radiation facility ELETTRA. Derived data of this study are available from the corresponding author C.P. on request.

## References

[1] Salanne M (2016). Efficient storage mechanisms for building better supercapacitors. Nat. Energy.

[2] Béguin F, Presser V, Balducci A, Frackowiak E (2014). Carbons and electrolytes for advanced supercapacitors. Adv. Mater..

[3] Forse AC, Merlet C, Griffin JM, Grey CP (2016). New perspectives on the charging mechanisms of supercapacitors. J. Am. Chem. Soc..

[4] Lin, Z. et al. Materials for supercapacitors: when Li-ion battery power is not enough. *Mater. Today* **21**, 419–436 (2018).

[5] Suss ME (2015). Water desalination via capacitive deionization: what is it and what can we expect from it?. Energy Environ. Sci..

[6] Griffin JM (2015). In situ NMR and electrochemical quartz crystal microbalance techniques reveal the structure of the electrical double layer in supercapacitors. Nat. Mater..

[7] Levi MD, Sigalov S, Aurbach D, Daikhin L (2013). In situ electrochemical quartz crystal admittance methodology for tracking compositional and mechanical changes in porous carbon electrodes. J. Phys. Chem. C.

[8] Levi MD, Salitra G, Levy N, Aurbach D, Maier J (2009). Application of a quartz-crystal microbalance to measure ionic fluxes in microporous carbons for energy storage. Nat. Mater..

[9] Prehal C (2015). Tracking the structural arrangement of ions in carbon supercapacitor nanopores using in situ small-angle X-ray scattering. Energy Environ. Sci..

[10] Prehal C (2017). Quantification of ion confinement and desolvation in nanoporous carbon supercapacitors with modelling and in situ X-ray scattering. Nat. Energy.

[11] Merlet C (2013). Highly confined ions store charge more efficiently in supercapacitors. Nat. Commun..

[12] Kondrat S, Kornyshev AA (2011). Superionic state in double-layer capacitors with nanoporous electrodes. J. Phys. Condens. Matter.

[13] Chmiola J (2006). Anomalous increase in carbon capacitance at pore sizes less than 1 nanometer. Science.

[14] Jäckel, N., Simon, P., Gogotsi, Y. & Presser, V. Increase in capacitance by subnanometer pores in carbon. *ACS Energy Lett.* **1**, 1262–1265 (2016).

[15] Richey FW, Dyatkin B, Gogotsi Y, Elabd YA (2013). Ion dynamics in porous carbon electrodes in supercapacitors using in situ infrared spectroelectrochemistry. J. Am. Chem. Soc..

[16] Prehal C (2017). A carbon nanopore model to quantify structure and kinetics of ion electrosorption with in situ small angle X-ray scattering. Phys. Chem. Chem. Phys..

[17] Sigalov S, Levi MD, Salitra G, Aurbach D, Maier J (2010). EQCM as a unique tool for determination of ionic fluxes in microporous carbons as a function of surface charge distribution. Electrochem. Commun..

[18] Tsai WY, Taberna PL, Simon P (2014). Electrochemical quartz crystal microbalance (EQCM) study of ion dynamics in nanoporous carbons. J. Am. Chem. Soc..

[19] Levi MD, Sigalov S, Salitra G, Aurbach D, Maier J (2011). The effect of specific adsorption of cations and their size on the charge-compensation mechanism in carbon micropores: the role of anion desorption. Phys. Chem. Chem. Phys..

[20] Griffin JM (2014). FD 176: ion counting in supercapacitor electrodes using NMR spectroscopy. Faraday Discuss..

[21] Deschamps M (2013). Exploring electrolyte organization in supercapacitor electrodes with solid-state NMR. Nat. Mater..

[22] Weingarth D (2014). Graphitization as a universal tool to tailor the potential‐dependent capacitance of carbon supercapacitors. Adv. Energy Mater..

[23] Barbieri O, Hahn M, Herzog A, Kötz R (2005). Capacitance limits of high surface area activated carbons for double layer capacitors. Carbon N. Y..

[24] Hahn M (2004). Interfacial capacitance and electronic conductance of activated carbon double-layer electrodes. Electrochem. Solid State Lett..

[25] Lu, M., Beguin, F. & Frackowiak, E. *Supercapacitors: Materials, Systems and Applications* 1–68 (John Wiley & Sons, Weinheim, Germany 2013).

[26] Bard, A. J. & Faulkner, L. R. *Electrochemical Methods: Fundamentals and Applications*. 2nd edn (John Wiley & Sons, New York, USA 2000).

[27] Laheäär A, Przygocki P, Abbas Q, Béguin F (2015). Appropriate methods for evaluating the efficiency and capacitive behavior of different types of supercapacitors. Electrochem. Commun..

[28] Biesheuvel PM, Porada S, Levi M, Bazant MZ (2014). Attractive forces in microporous carbon electrodes for capacitive deionization. J. Solid State Electr..

[29] Pell WG, Conway BE, Marincic N (2000). Analysis of non-uniform charge/discharge and rate effects in porous carbon capacitors containing sub-optimal electrolyte concentrations. J. Electroanal. Chem..

[30] Härtel A, Janssen M, Weingarth D, Presser V, van Roij R (2015). Heat-to-current conversion of low-grade heat from a thermocapacitive cycle by supercapacitors. Energy Environ. Sci..

[31] Forse AlexanderC (2017). Direct observation of ion dynamics in supercapacitor electrodes using in situ diffusion NMR spectroscopy. Nat. Energy.

[32] Prehal, C. *Ion Electrosorption in Nanoporous Carbons*. Doctor thesis, Montanuniversität Leoben (2017).

[33] Pedersen J (2004). A flux- and background-optimized version of the NanoSTAR small-angle X-ray scattering camera for solution scattering. J. Appl. Crystallogr..

[34] Gor GY, Thommes M, Cychosz KA, Neimark AV (2012). Quenched solid density functional theory method for characterization of mesoporous carbons by nitrogen adsorption. Carbon N. Y..

[35] Chantler, C. T. et al. *X-Ray Form Factor, Attenuation and Scattering Tables (version 2.1)*, http://physics.nist.gov/ffast (1995, 1996, 2001).

[36] Nightingale ER (1959). Phenomenological theory of ion solvation. effective radii of hydrated ions. J. Phys. Chem..

[37] Koneshan S, Rasaiah JC, Lynden-Bell RM, Lee SH (1998). Solvent structure, dynamics, and ion mobility in aqueous solutions at 25 °C. J. Phys. Chem. B.

