# Author Correction: Salt concentration and charging velocity determine ion charge storage mechanism in nanoporous supercapacitors

**DOI:** 10.1038/s41467-019-08310-1

**Published:** 2019-01-11

**Authors:** C. Prehal, C. Koczwara, H. Amenitsch, V. Presser, O. Paris

**Affiliations:** 1 Institute of Physics, Montanuniversitaet Leoben, Franz-Josef Straße 18, 8700 Leoben, Austria; 2 Institute of Inorganic Chemistry, Graz University of Technology, Stremayrgasse 9/IV, 8010 Graz, Austria; 3 INM-Leibniz Institute for New Materials, Campus D2 2, 66123 Saarbrücken, Germany; 4 Department of Materials Science and Engineering, Saarland University, Campus D2 2, 66123 Saarbrücken, Germany; 5 Present Address: Institute for Chemistry and Technology of Materials, Graz University of Technology, Stremayrgasse 9/V, 8010 Graz, Austria

Correction to: *Nature Communications*; 10.1038/s41467-018-06612-4; published online 08 October 2018

The original version of this Article contained an error in the Acknowledgements, which was incorrectly omitted from the end of the following: ‘The research leading to these results has received funding from the European Community’s Horizon 2020 Framework Programme under grant agreement nº 730872.’ This has been corrected in both the PDF and HTML versions of the Article.

