# Supplementary material for: Salt concentration and charging velocity determine ion charge storage mechanism in nanoporous supercapacitors
Source: Nat Commun. 2018 Oct 8;9:4145. doi: 10.1038/s41467-018-06612-4 (PMC6175899; doi:10.1038/s41467-018-06612-4)
Supplement: Supplementary file 1 — Supplementary Information [file 41467_2018_6612_MOESM1_ESM.pdf]

Supplementary Information

Salt concentration and charging velocity determine ion charge storage mechanism in nanoporous supercapacitors

C. Prehal[1,†,*], C. Koczwara[1], H. Amenitsch[2], V. Presser,[3,4,]O. Paris[1,*]

1. Institute of Physics, Montanuniversitaet Leoben, Franz-Josef Straße 18, 8700 Leoben, Austria
2. Institute of Inorganic Chemistry, Graz University of Technology, Stremayrgasse 9/IV, 8010 Graz, Austria
3. INM - Leibniz Institute for New Materials, Campus D2 2, 66123 Saarbrücken, Germany
4. Department of Materials Science and Engineering, Saarland University, Campus D2 2, 66123 Saarbrücken, Germany

† Current address

Institute for Chemistry and Technology of Materials, Graz University of Technology, Stremayrgasse 9/V, 8010 Graz, Austria

* Corresponding author's eMail: christian.prehal@tugraz.at, oskar.paris@unileoben.ac.at

Supplementary Figures

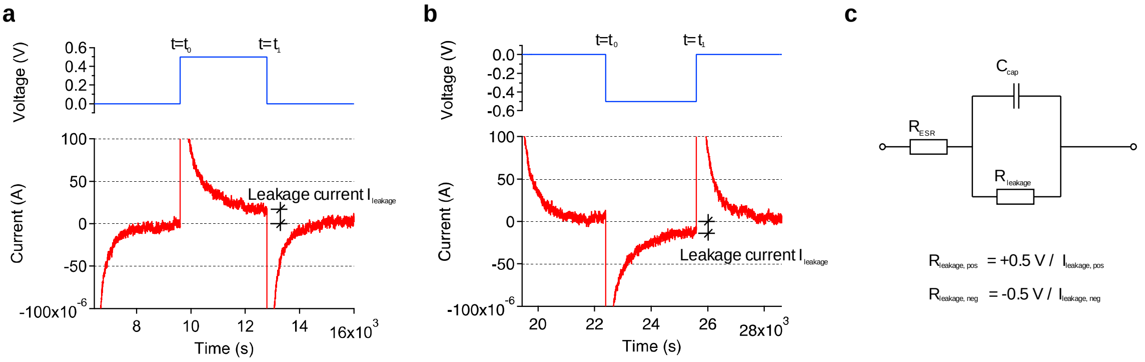

Supplementary Figure 1: Cell voltage (blue) and current (red) versus time for a chronoamperometry step at -0.5 V (a) and at +0.5 V (b), shown exemplarily for 0.01M RbBr. The constant current after equilibration at non-zero voltages is attributed to the leakage current. Panel (c) shows an equivalent circuit model for the supercapacitor.

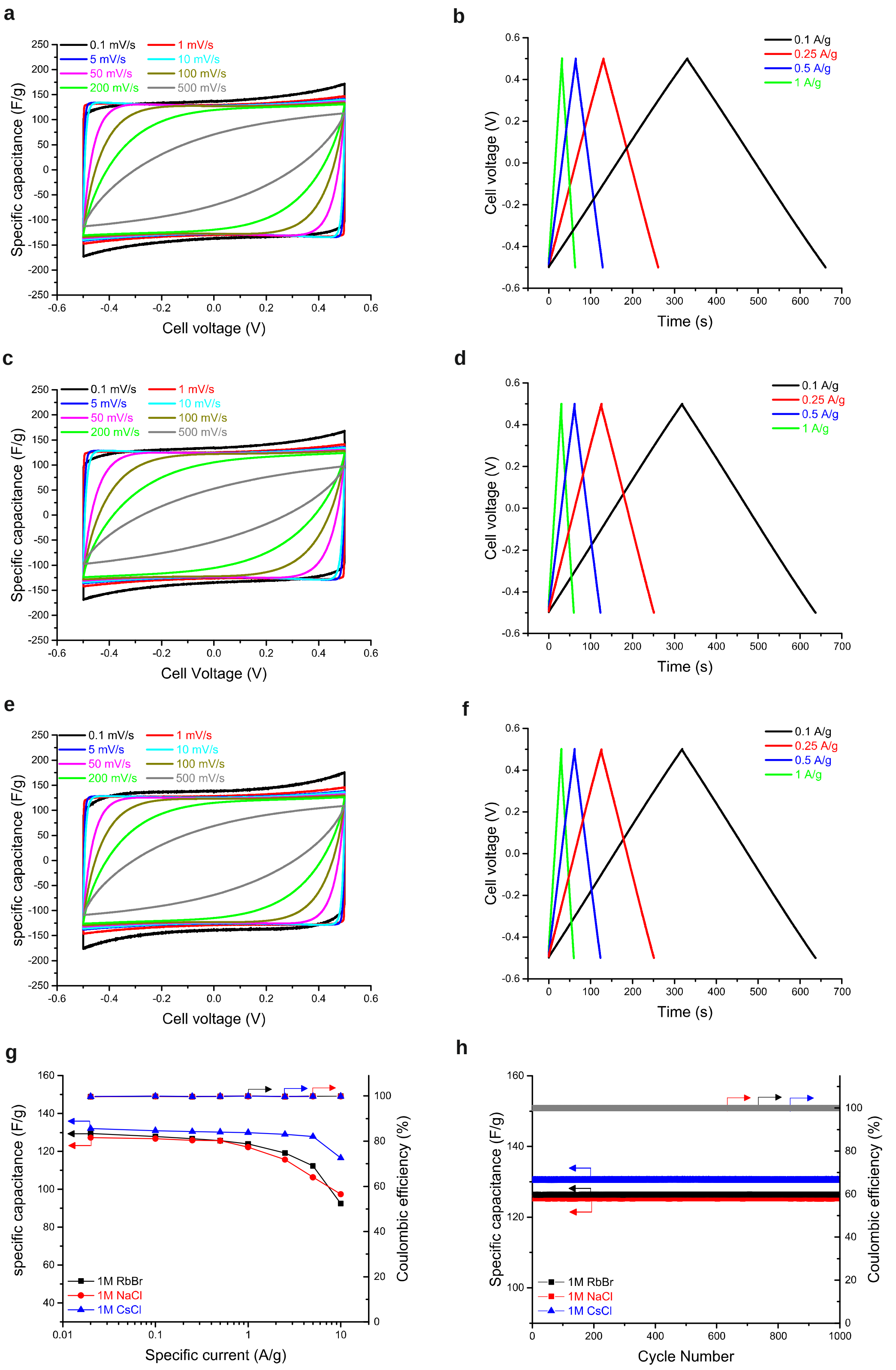

Supplementary Figure 2: CV measurements using the activated carbon AC as electrode material and 1M CsCl (a), 1M NaCl (c), 1M RbBr (e) aqueous electrolytes with scan rates from 0.1 to 500 mV/s. GCPL measurements 1M CsCl (b), 1M NaCl (d) and 1M RbBr (f) with specific currents from 0.1 to 1 A/g. Power handling and coulombic efficiency for specific currents from 0.02 to 10 A/g (g). Cyclic stability test for 1000 cycles using a specific charging and discharging current of 1 A/g (h)

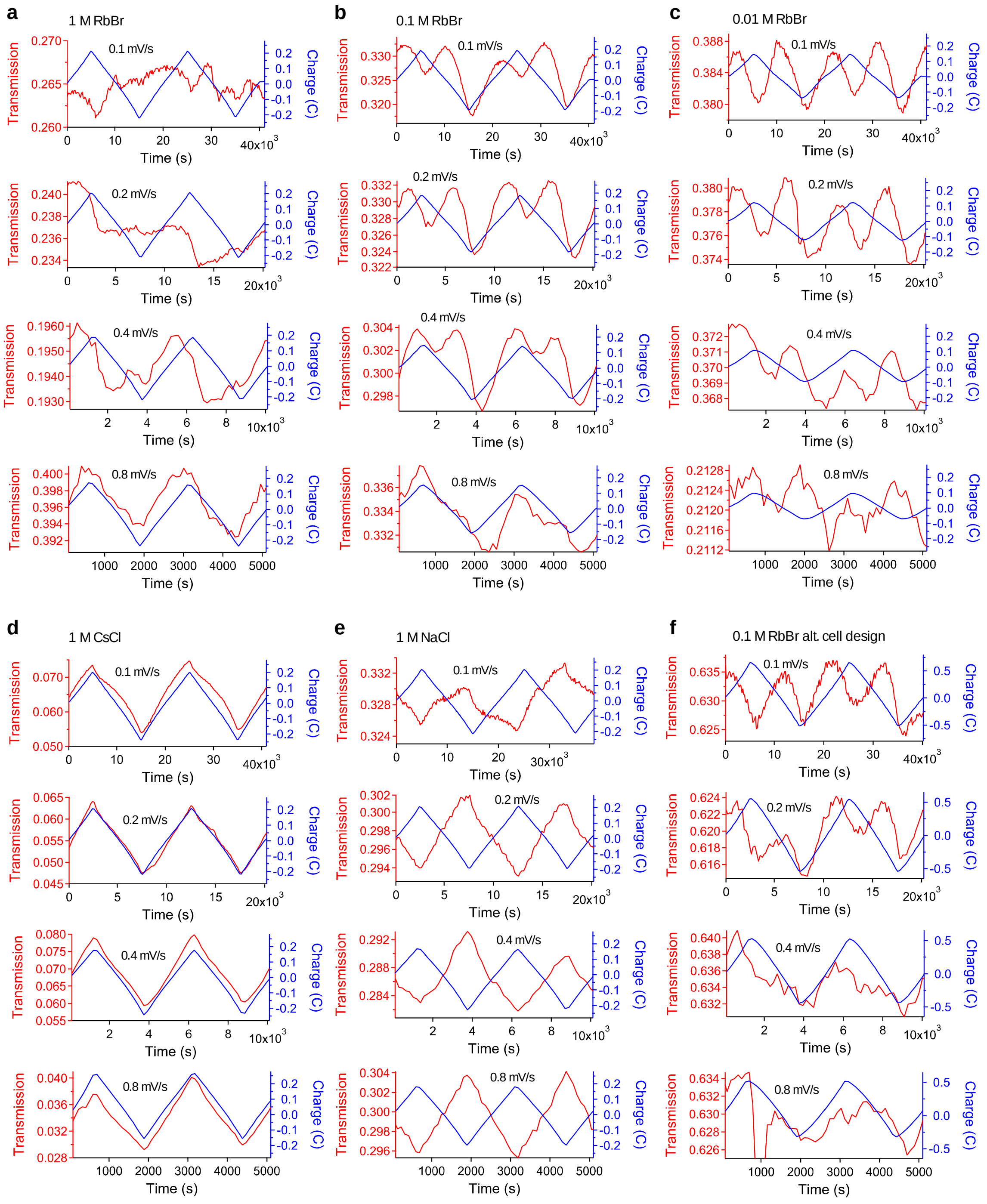

Supplementary Figure 3: (a-f) Transmission (red) and electrode charge (blue) vs. time for three cyclic voltammetry periods at 0.1, 0.2, 0.4 and 0.8 mV/s scan-rate. The transmission data is averaged over two CV periods and the relative attenuation (**Eq. 2**) vs. charge (**Fig. 2**) plotted accordingly. The electrode charge is obtained by integrating the current (corrected for leakage currents) over time, where a long enough equilibration time at 0V is required before starting the CV measurements.

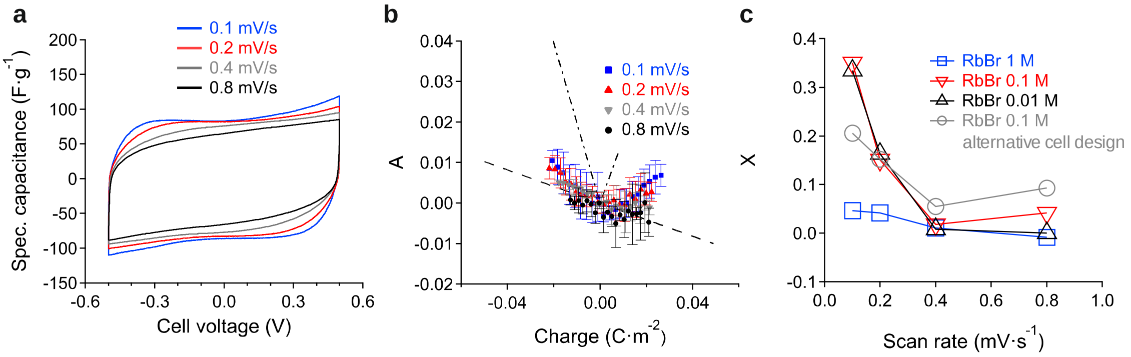

Supplementary Figure 4: Cyclic voltamogram (a) and relative attenuation A vs. charge (b) of a 0.1 M RbBr electrolyte tested in an in situ cell using the same WE material but a different ratio between WE micropore volume and bulk electrolyte volume than the conventional assembly in the main paper. Charge mechanism parameter X vs. scan rate (c) for cells with conventional design (blue, red, black) and alternative cell design with reduced micropore to bulk electrolyte volume ratio (grey).

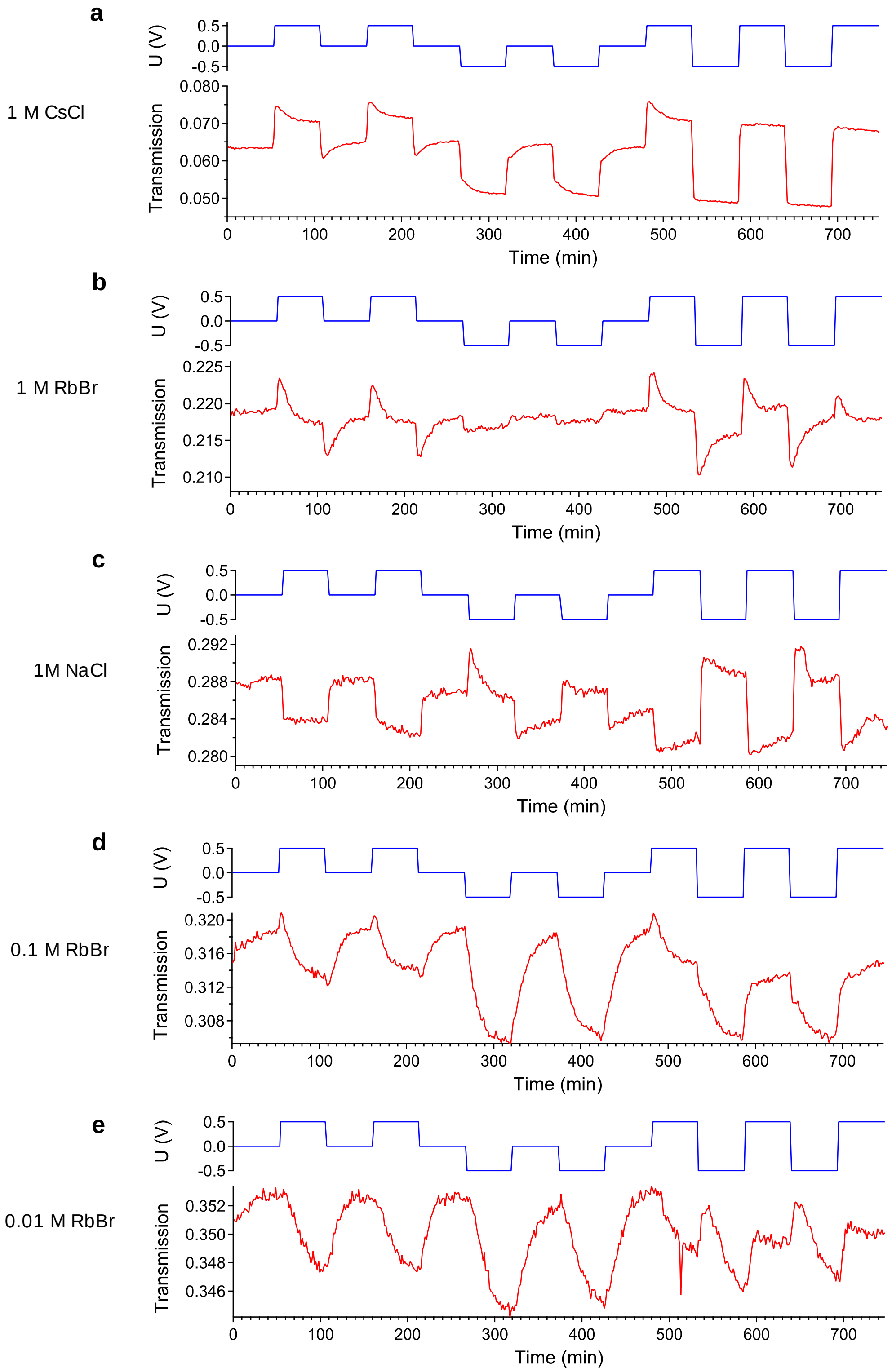

Supplementary Figure 5: X-ray transmission (red) and applied voltage (blue) versus time for chronoamperometry measurements of in situ cells using the AC working electrode and 1 M CsCl (a), 1 M RbBr (b), 1 M NaCl (c), 0.1 M RbBr, (d) and 0.01 M RbBr (e).

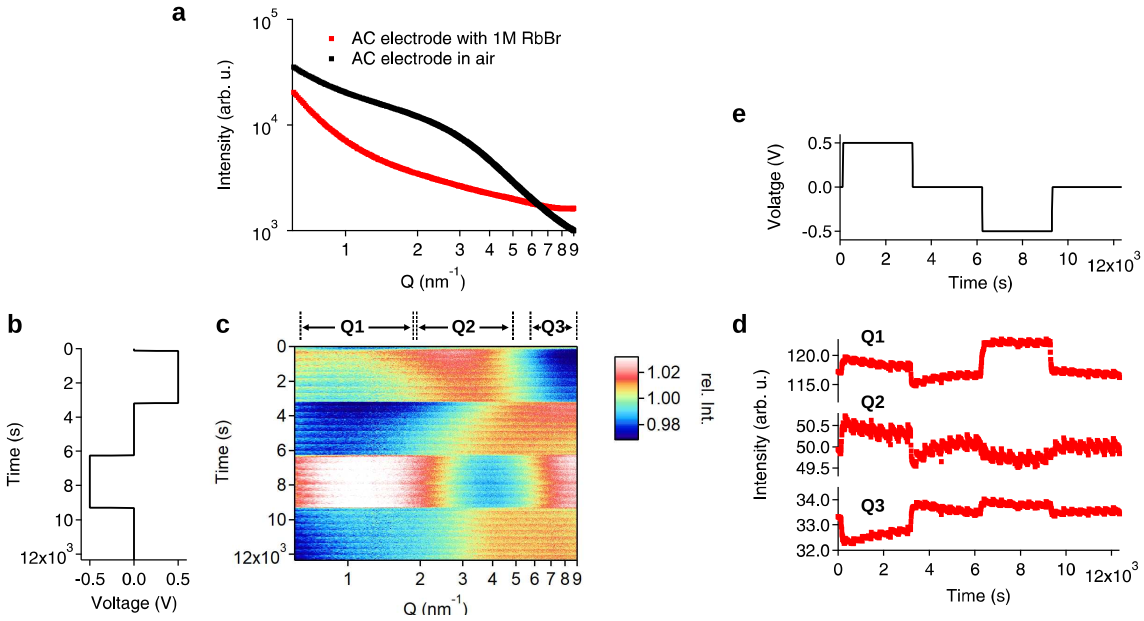

Supplementary Figure 6: (a) SAXS intensity versus scattering vector length Q for the empty AC electrode in air (black) and infiltrated with 1 M RbBr (red). The relative SAXS intensity change (SAXS intensity at a given time t divided by the intensity at t=0) as a function of time or voltage (b) is given in (c). The average SAXS intensity in three different Q-regimes is given as a function of time (d).

Supplementary Notes

Supplementary Note 1

Leakage current subtraction. Since leakage currents for CVs at scan rates smaller than 1 mV/s are not negligible in aqueous systems, they were determined using CA measurements and subtracted accordingly (Supplementary Fig. 1a-b). We make here the reasonable assumption that the leakage currents for positive and negative polarization correspond to the stable current measured after charging for a long time (here approximately 1 hour). Assuming an equivalent circuit model (Supplementary Fig. 1c), $R_{leakage}$ can be calculated using the applied voltage and the measured leakage current via $R_{leakage} = U/I_{leakage}$. The pure capacitance values C_{cap} for this system can thus easily be calculated by $C_{cap} = \frac{Q_{cap}(t=t_1)}{U_{cap}(t=t_1)} = \frac{\int_0^{t1}(I_{cap}-I_{leakage})dt}{U_{cell}}$, where $U_{cap} = U_{cell}$ at the end of the charging process. For the shown cell C_{cap} equals 169 F/g and 153 F/g for positive and negative polarization, respectively. However, more appropriately the specific capacitance needs to be evaluated from GCPL measurements as shown above (Supplementary Fig. 2).

Supplementary Note 2

Electrochemical characterization. The electrochemical performance of the AC electrode material with the three electrolytes (1M CsCl, 1M RbBr, 1M NaCl) is characterized in a custom-built cell optimized for performance testing of supercapacitors (Supplementary Fig. 2). All measurements were carried out on a Gamry Reference 600 potentiostat using a symmetric two-electrode setup. The custom-built cell made of a polyether ether ketone (PEEK) housing has spring loaded titanium pistons for electrical contact. The actual supercapacitor cell is a multilayer assembly with two platinum current collectors, two electrodes with a diameter of 12 mm, and a thickness of 200 µm and a glass fiber separator (Whatman GF/A) in-between the electrodes.

Cyclic voltammetry (CV) at different scan rates (Fig. 1a,c,e) and galvanostatic charge and discharge with potential limitation (GCPL; Supplementary Fig. 2b,d,f) reveal a similar behavior for all three electrolytes. The capacitance of the entire cell $C_{cell}$was calculated via $C_{cell} = 2E/{U_{max}}^2$ with the energy E being defined as $E = I\int_{t_1}^{t_2} U(t)dt$. To calculate the specific capacitance of a single electrode C_{el} a factor of 4 has to be considered: $C_{el} = \frac{2C_{cell}}{0.5\,m}$, where m is the total mass of both electrodes.[1] For smaller specific currents, the specific capacitance for each electrolyte is practically the same; only for currents much higher than typical currents in the in situ experiment some deviations are present (Supplementary Fig. 2g). The good capacitance retention, even at high specific currents, can be attributed to the optimized activated carbon electrodes, the high ionic mobilities in aqueous electrolytes, and the minimized serial resistance in the custom-built supercapacitor cells. At a current of 0.1 A/g the specific capacitance of CsCl, RbBr and NaCl amounts to

130 F/g, 128 F/g and 127 F/g, corresponding to energy densities of 4.51 Wh/kg, 4.44 Wh/kg and 4.41 Wh/kg, respectively.

The power handling test was carried out for currents ranging from 0.02 to 10 A/g. The cyclic stability was tested over 1000 cycles using a specific current of 1 A/g. As the specific current for the charging and discharging processes was similar, the coulombic efficiency was calculated by $\frac{t_d}{t_c}$, where t_c is the charging- and t_d the discharging time. As shown in Supplementary Fig. 2h, both specific capacitance and coulombic efficiency are stable during the 1000 cycles tested.

Note that the increased ion diffusion pathways due to the specific design of the in situ cell assembly increases the equivalent serial resistance compared to the cells used for performance testing shown in Supplementary Fig. 2.

Supplementary Note 3

Selection of electrolytes. As stated in the main manuscript and shown in Supplementary Fig. 2 cells with 1 M CsCl, 1 M RbBr, and 1 M NaCl show very similar electrochemical response (Fig. 1a,d,e), but a notably different X-ray attenuation behavior (Fig. 2a-c). These salts having different ratios of cation and anion attenuation coefficients (Table 1) enable a consistent interpretation of the experimental data using Eq. 2 (see also Ref. [2])". Thus, the particular selection of electrolytes is based on experimental deliberations only. According to Table 1, the ratio between cation and anion X-ray attenuation coefficients of the three salts varies in a systematic manner. While the X-ray attenuation coefficient of Cs^+ is by far larger than the Cl^- attenuation coefficient, for NaCl the anion attenuation coefficient is larger than the cation one. In the case of RbBr the attenuation coefficients of cations and anions are very similar. For pure ion swapping, where the total ion concentration remains constant during charging and discharging, the transmission and relative attenuation of the RbBr electrolyte would therefore remain nearly constant during charging. These relations are reflected in the relative attenuation vs. charge curves in Fig. 2a-c. Since the electrochemical behavior is practically identical (differences regarding hydrated, non-hydrated ion size or hydration energy obviously play a minor role), no significant differences regarding cation and anion concentration changes are expected. Therefore, using these three different electrolytes facilitates a consistent interpretation of the experimental data in a so called "contrast variation" experiment.

Due to the comparable X-ray attenuation coefficients of cations and anions in the case of RbBr, the relative attenuation would increase equally for both, positive and negative polarization. For this reason, RbBr is best suited (most sensitive) for the interpretation of the X-ray transmission data regarding ion charge storage mechanisms. Using RbBr for the analysis of low molar electrolytes produces the most significant differences between Figs. 2d-f and therefore keeps the experimental error low.

In summary, although the experimental data (Fig. 2 and Fig. 3) of the 1 M electrolytes look different (due to different X-ray attenuation coefficients) ion charge storage

mechanisms of the three electrolytes are, within the experimental error, identical (apart from deviations of RbBr in Fig. 3, discussed in the last part of the main paper).

Supplementary Note 4

Ion concentration change with alternative cell assembly. To study a possible dependence of the charging mechanism on the specific design of an asymmetric supercapacitor cell, the volume of the bulk electrolyte reservoir was lowered using an alternative cell assembly with 0.1 M RbBr. In contrast to the conventional design instead of 5 separators only a single Whatman (GF/A) glass fiber separator was used. In addition, the WE mass was increased (13.8 mg, diameter of 12 mm) to further decrease the ratio between the "active" WE micropore volume and bulk electrolyte volume (CE capacitance oversized by a factor of three). Considering the 5-fold increased separator volume and 5-fold increased WE volume the micropore to bulk electrolyte volume ratio should be decreased by a factor of 25 (as upper limit) in the alternative cell assembly.

CVs of this alternative cell assembly using the same WE material and 0.1 M RbBr, are shown in Supplementary Fig. 4a. The apparently lowered gravimetric capacitance compared to Fig. 1b is caused by the small ratio of WE to CE capacitance (CE oversized only by a factor of three).

Supplementary Fig. 4b-c shows that the charging mechanism parameter X depends also on the cell design. The scan-rate, at which the transition from counter-ion adsorption towards ion swapping takes place, depends on the specific cell design. As compared to the cell assembly with large bulk electrolyte volume, counter-ion adsorption seems to be suppressed in this case (compare Fig. 2e with Supplementary Fig. 4b-c).

Possibly, cells with a small electrolyte reservoir (i.e., small bulk electrolyte volume) show preferentially ion swapping. In this case, counter-ions adsorbing in the WE pores during charging need to be provided mainly from the CE (where the same ions desorb as co-ions). Since the same process takes place for ions with opposite sign, counter-ions adsorb and co-ions desorb roughly with the same rate (neglecting the differences in the mobilities of the different ion species). In the case of counter-ion adsorption the total ion concentration in both electrodes increase, that is, the concentration within the bulk electrolyte decreases. In equilibrium, the electrochemical potential of micropores and bulk electrolyte are equal. Since ions are depleted in the bulk electrolyte reservoir during counter-ion adsorption the chemical potential of the bulk electrolyte is expected to change to a larger extend if the electrolyte reservoir is small. However, the chemical potential of the bulk electrolyte (entropic contribution) can be lowered only to a certain extend (see theoretical work for CDI, such as Ref.[3]).

Supplementary Note 5

In situ SAXS. In situ SAXS measurements were carried out to confirm that the changes monitored by XRT occur within the micropores. The data were treated and visualized as

described in recent studies (Ref.[2,4,5]). Relative SAXS intensity changes in different Q-regimes (scattering vector length Q) contain information regarding local structural rearrangements or global ion concentration changes. Following the SAXS interpretation of our recent work, the average intensity change in the Q-regime Q-2 mainly reflects mean changes in the SAXS contrast between carbon matrix and in-pore electrolyte. It therefore contains information about ion concentration changes within the micropores and is inversely related to the attenuation signal in Fig. 3. An inverse relation between Supplementary Fig. 6d (Q-2) and the relative attenuation in Fig. 3b confirms that basically all ion concentration changes discussed on basis of XRT measurements indeed take place within the micropores.

The intensity maximum at negative polarization in the Q-regime Q-1 (Supplementary Fig. 6c-d) can be related to the local structural rearrangement of counter-ions into pore sites with high degree of confinement[5]. Since this maximum remains constant after a short increase during negative polarization, we conclude that the local ion rearrangement takes place as a direct response to the applied electrode charge and happens on the same timescale as the charge accommodation. Further investigations are required to verify whether the (neutral) increase of the total ion concentration after the actual charging has no effect on the local ion arrangement at all.

Supplementary References

1 Laheäär, A., Przygocki, P., Abbas, Q. & Béguin, F. Appropriate methods for evaluating the efficiency and capacitive behavior of different types of supercapacitors. *Electrochem. Commun.* **60**, 21-25 (2015).

2 Prehal, C. *et al.* Tracking the structural arrangement of ions in carbon supercapacitor nanopores using in situ small-angle X-ray scattering. *Energy Environ. Sci.* **8**, 1725-1735 (2015).

3 Biesheuvel, P. M., Porada, S., Levi, M. & Bazant, M. Z. Attractive forces in microporous carbon electrodes for capacitive deionization. *J. Solid State Electr.* **18**, 1365-1376 (2014).

4 Prehal, C. *et al.* A carbon nanopore model to quantify structure and kinetics of ion electrosorption with in situ small angle X-ray scattering. *Phys. Chem. Chem. Phys.* **19**, 15549 (2017).

5 Prehal, C. *et al.* Quantification of ion confinement and desolvation in nanoporous carbon supercapacitors with modelling and in situ X-ray scattering. *Nat. Energy* **2**, 16215 (2017).
